# Retrospective Study on Optimizing Breast Augmentation Outcomes in Transgender Patients: A Comprehensive Analysis of Tumescent Local Anesthesia Technique

**DOI:** 10.1007/s00266-024-03922-5

**Published:** 2024-03-08

**Authors:** Matilde Tettamanzi, Federico Ziani, Manuela Rodio, Giovanni Arrica, Giovanni Muratore, Anna Manconi, Claudia Trignano, Edoardo Beatrici, Corrado Liperi, Corrado Rubino, Emilio Trignano

**Affiliations:** 1 Plastic Surgery Unit, Department of Surgical, Microsurgical and Medical Sciences, University of Sassari, Sassari, Italy; 2 Department of Biomedical Sciences, University of Sassari, Sassari, Italy; 3 Department of Urology, Humanitas Research Hospital – IRCCS, Milan, Italy; 4 Intensive Care Unit, Emergency Department, AOU Sassari, Sassari, Italy

**Keywords:** Transgender, Tumescent local anesthesia, Breast, Breast augmentation, Mammoplasty, Breast implants

## Abstract

**Background:**

Tumescent local anesthesia (TLA) involves the infusion of a saline solution containing lidocaine and epinephrine into tissues to achieve localized anesthesia and vasoconstriction. While the use of TLA in sub-glandular augmentation mammoplasty has been documented, we present a modified TLA approach for primary sub-muscular breast augmentation in transgender patients based on our experience over the past years.

**Methods:**

Between the years 2014 and 2021, we performed primary sub-muscular breast augmentation on 20 transgender patients under TLA and conscious sedation. The tumescent solution consisted of 25 mL of 2% lidocaine, 8 mEq of sodium bicarbonate, and 1 mL of epinephrine (1 mg/1 mL) in 1000 mL of 0.9% saline solution. Initially, the solution was infiltrated between the pectoral fascia and the mammary gland, and subsequently, during the surgery, under the pectoralis major muscle.

**Results:**

The average volume of tumescent solution infiltrated during TLA was 740 mL per breast. There were no reports of adrenaline or lidocaine toxicity, and no cases required a conversion to general anesthesia. Patients experienced no pain or discomfort during the preoperative infiltration or surgical procedure.

Reoperations due to short-term complications never occurred. We observed a major complication rate of 5%, represented by 1 hematoma. Long-term complications comprised one case of implant dislocation and one occurrence of dystrophic scar formation. No cases of capsular contracture needing reoperation, asymmetry, and implant rupture occurred. In total, one individual (5%) requested larger implants. Follow-up time ranged from 30 days to 1 years.

**Conclusions:**

Overall, augmentation mammaplasty is a valuable choice for transgender women aiming to enhance their feminine characteristics and alleviate gender dysphoria. It is imperative for patients to conduct thorough research, grasp the potential pros and cons, and consult experienced healthcare professionals in transgender care. Additionally, tumescent local anesthesia (TLA) has proven to be a safe and efficient method for sub-muscular breast augmentation, providing effective pain control with minimal postoperative complications, resulting in high patient satisfaction.

**Level of Evidence IV:**

This journal requires that authors assign a level of evidence to each article. For a full description of these Evidence-Based Medicine ratings, please refer to the Table of Contents or the online Instructions to Authors www.springer.com/00266.

## Introduction

Augmentation mammaplasty, commonly known as breast augmentation, is a surgical procedure that involves increasing the size and enhancing the shape of the breasts. It is one of the most popular procedures in esthetic surgery even if its scenario has changed a lot over years. It is a well-established procedure typically performed on cisgender women who desire larger or more shapely breasts. However, in recent years, it has also become increasingly common among transgender patients (assigned male at birth, but identify as female) who wish to develop more feminine characteristics, including breast enlargement. For transgender patients, breast augmentation can be an essential aspect of their gender-affirming journey, as it helps to align their physical appearance with their gender identity. Before proceeding with augmentation mammaplasty, transgender patients typically undergo hormone replacement therapy (HRT) to induce feminizing effects, which may lead to some natural breast growth [1]. However, HRT results can vary from person to person, and some individuals may not achieve their desired breast size through hormones alone. In such cases, breast augmentation can provide a more predictable and substantial increase in breast size [2]. At surgery, the choice of local or general anesthesia depends on many factors, but essentially the invasiveness and the risk of the surgical procedure and the preferences of patient and surgeon. The use of tumescent local anesthesia (TLA) is a technique developed in several surgical procedures but mainly in liposuction; it consists of infiltration of large volumes of saline solution with lidocaine and epinephrine in the surgical field [3, 4]. In augmentation mammoplasty, TLA has been described and is a useful technique, shortening the time of surgery, facilitating the dissection, and reducing bleeding and postoperative pain [5, 6]. In this article, we describe the use of TLA for primary transgender breast augmentation, reporting our cumulative experience during the past 7 years.

## Methods

**Fig 1:**
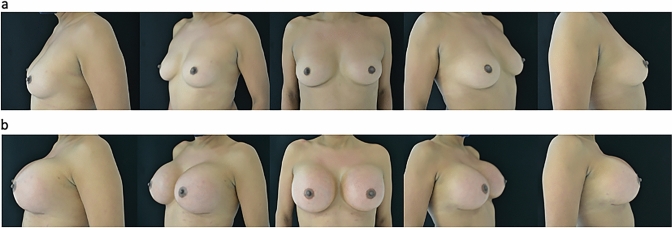
**a** Preoperative view. **b** Postoperative view after 1 year

**Fig 2:**
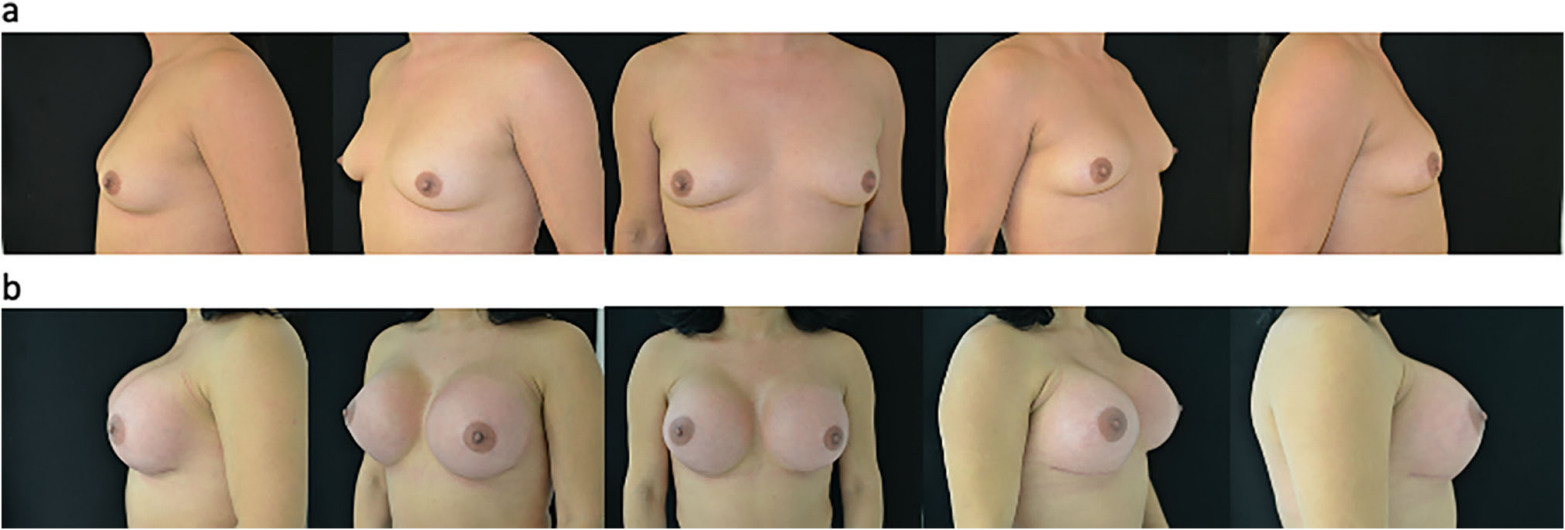
**a** Preoperative view. **b** Postoperative view after 6 months

From 2014 to 2021, a cohort of 20 transgender individuals underwent bilateral primary breast augmentation procedures. All surgical interventions were conducted within the confines of an accredited outpatient healthcare facility. The surgical team consisted of a board-certified plastic surgeon, an assistant surgeon, an operating room nurse, and a board-certified anesthesiologist. The mean age of the patients was 36 years, with a range spanning from 28 to 49 years, while the mean body weight and body mass index (BMI) averaged at 81.8 kg and 27.3 kg/m^2^, respectively. The prospective patients received comprehensive information concerning the utilization of implant-based breast surgery, its clinical indications, and potential complications, such as implant-related infections and postoperative hemorrhage. Preoperative assessments encompassed standard hematological evaluations, cardiac examinations, and breast imaging via ultrasound and/or mammography. Each patient satisfied the American Society of Anesthesiologists’ (ASA) criteria for status I or II, while exclusion criteria encompassed an ASA status of III or higher and a body mass index (BMI) exceeding 35 (Table 1). The breast implants utilized in these procedures were characterized by their silicone gel composition, microtextured silicone surface and round shape, specifically sourced from Nagor and Motiva (Nagor Limited, Isle of Man, UK; Motiva European Distribution Center, Wommelgem, Belgium). Prior to the surgical intervention, implant dimensions and shapes were selected with consideration of the patient’s breast dimensions and anterior thoracic wall size. Additionally, patients had the opportunity to try implant sizers while wearing a sports brassiere in front of a mirror to facilitate optimal implant volume selection. To minimize the risk of complications related to platelet clotting, medications that could influence this process were discontinued 5–7 days prior to the surgical intervention or substituted with acceptable alternatives. Preoperative markings were executed while the patient maintained an upright position; midsternal line, submammary fold, breast width, projection of the submammary fold on the midline, and lateral thoracic borders are drawn. Photographic documentation was acquired before the patient entered the OR and was positioned supine on the operating table. Peripheral intravenous access was established for each patient, and vital signs were continuously monitored throughout both the surgical procedure and the postoperative recovery period. Breast anesthesia encompassed two distinct phases: one prior to incision and the other following the exposure of the fascia of the pectoralis major muscle. A tumescent solution was prepared by mixing 20 mL of 2% lidocaine, 8 mEq of sodium bicarbonate, and 1 mL of epinephrine (1 mg/1 mL) within 1000 mL of 0.9% saline solution, being the lidocaine standard dose from 1.4 to 2.8 mg/kg [5]. Approximately 700–780 mL of this solution was introduced into each breast. The cutaneous incision site was infiltrated with a solution containing 1% lidocaine and 1:100,000 epinephrine. During the initial anesthesia phase, the plane above the superficial fascia of the pectoralis major muscle was identified by palpation, and a spinal needle was introduced and connected to a peristaltic infiltration pump. The infusion was halted when the tissue became engorged and vasoconstricted. In this series of cases, the average volume of tumescent solution infiltrated was 740 mL per breast. The volume of solution infiltrated varied according to the size of the breast and the patient’s BMI. Infiltration ensured comprehensive anesthesia through direct contact. A 5-cm skin incision was performed in the submammary sulcus approximately 40 min later to allow time for the epinephrine and lidocaine to take effect. Following the exposure of the fascia of the pectoralis major muscle, it was infiltrated with 1 mL of 1% lidocaine with 1:100,000 epinephrine, and a blunt multi-fenestrated cannula with a 2 mm diameter was introduced and left in place within the muscle and secured with a single 4–0 silk round block suture. Subsequently, a volume of 240 mL of tumescent solution was injected using a luer-lock syringe. Upon completion of the same procedure in the contralateral breast, the muscle incision was carried out using electrocautery, and dissection continued until the pectoralis major muscle was released entirely in the inferior and medial directions up to the superior extent of the areola (fourth–sixth rib), and superiorly up to the anatomical border of the pectoralis. Progressive blood vessel coagulation was performed during pocket dissection to prevent post-clearance bleeding, consequent to the vasoconstrictive effects of adrenaline. A large size fiberoptic retractor with smoke evacuation capabilities was employed during pocket dissection. Prior to implant placement, sterile drapes and gloves were changed, and instruments were disinfected with chlorhexidine; subsequently, the pocket was irrigated with a 50% diluted hydrogen peroxide solution, followed by a saline solution, and finally, a gentamicin solution. Surgical drains were not utilized, and wound closure was executed using monocryl 3–0 and 4–0 absorbable sutures at the level of the fascia, subcutaneous and cutaneous tissue, with a sterile dressing applied to cover the incision site. Postoperatively, patients wore a snug sports brassiere for one month. After a 4-h observation period, patients were discharged. Depending on the individual’s allergy status, an oral antibiotic regimen (amoxicillin 875 mg/clavulanic acid 125 mg or ciprofloxacin 500 mg twice daily) was prescribed for a duration of 5 days and analgesia regimen with Toradol 15 mg every 12 h and Paracetamol 1gr if needed maximum 3/die. Subsequent postoperative follow-up appointments were scheduled for 1 day, 1 week, 2 weeks, 1–3–6 months, and 1 year (Figs. 1, 2), and during the visits, surgical healing, swelling, symmetry, and aesthetic outcomes are evaluated.

**Table 1:** Patients and surgery’s characteristics and demographic data

Characteristic	Values
Mean age	36 years
BMI	27.3 kg/m^2^
Mean body weight	81.8 kg
Average volume of TLA per breast	740 mL (range 700–780 mL)
Average time interval from solution infiltration to skin incision	40 min
Implant sizes	From 650 to 1000 cc
Mean duration of surgery	1 h and 40 min

## Results

Over the course of a 7-year interval, a comprehensive analysis was conducted on a cohort comprising 20 transgender individuals who had undergone sub-muscular breast augmentation procedures, exclusively employing the Tumescent Local Anesthesia technique. A mean volume of 740 mL of tumescent solution was infiltrated, with a range between 700 and 780 mL, and no instances of adverse effects related to adrenaline or lidocaine toxicity were documented. General anesthesia conversion was never necessitated during any of the procedures. The average time interval from solution infiltration to the initiation of skin incision was determined to be 40 min, a parameter determined through collaborative assessment by the surgical team and anesthesiologist. Departing from this time threshold resulted in heightened patient discomfort, while exceeding it conferred no discernible benefits to the patient. During the surgical intervention, no reports of pain emerged during the skin incision or the elevation of the major pectoralis muscle. The spectrum of implant sizes utilized ranged from 650 to 1000 cc. The mean duration of surgery employing the TLA technique encompassed 1 h and 40 min, incorporating bilateral infiltration, waiting periods, and the surgical procedure until completion. Within the scope of postoperative complications, comprising 5% of cases, one incident of hematoma was recorded. A minor complication rate of 10% was observed, characterized by one occurrence of implant dislocation and one instance of dystrophic scarring (as delineated in Table 2). Capsular contraction was evaluated according to the Baker classification [7]. At the 1-year mark, 10% of patients exhibited Baker grade I capsular contracture, while 5% displayed grade II; grade III or IV contractures were not observed. Patients expressed overall satisfaction with the TLA procedure, reporting no discomfort during the preoperative infiltration or throughout the entire surgical process. Most patients conveyed contentment with the aesthetic outcomes at the 1-year follow-up. A satisfaction survey was administered 3 months post-surgery, enabling patients to rate their pain management and satisfaction with their aesthetic results on a scale ranging from “unsatisfactory” to “excellent.” The majority of patients expressed high levels of satisfaction, only one patient requested larger implants, and there has been no need to reoperate. In one case, it has been necessary to increase the size of the breast by adding a lipofilling procedure to enhance the aesthetic result [8]. Importantly, all patients were subject to a follow-up period exceeding one year.

**Table 2:** Postoperative complication rate after sub-muscular breast augmentation in 20 transgender patients

Complications	Patients	%
Hematoma	1	5
Seroma	0	0
Implant dislocation	1	5
Dystrophic scar	1	5
Need for reintervention	0	0

## Discussion

In this article, we present our experience of 20 consecutive cases of TLA sub-muscular breast augmentation in transgender patients over a 7-year period time. The study’s detailed investigation into a modified tumescent local anesthesia approach for primary sub-muscular breast augmentation in transgender patients represents a significant stride in surgical practices catering to this specific demographic. The research highlights the feasibility and safety of TLA in this context, elucidating its potential to minimize complications and reduce patient discomfort during the preoperative phase and the surgical procedure itself. The absence of toxicity-related incidents from the infused tumescent solution and the ability to forego general anesthesia showcases the viability of this method, underscoring its potential as a preferable alternative in surgical settings [9]. Sub-muscular breast augmentation with the tumescent local anesthesia technique is very similar in transgender and cisgender patients, overall. Despite various authors recounting their experiences with different types of local anesthesia, contemporary breast augmentation procedures are predominantly conducted under general anesthesia. In a study conducted by Chung et al. [10], a target-controlled infusion (TCI) system was employed for intravenous anesthesia using a combination of propofol and remifentanil in conjunction with local anesthesia. The local anesthesia involved a blend of epinephrine, lidocaine, and ropivacaine. The authors administered an intercostal nerve block, covering the area from the second to the eighth intercostal nerve on the mid-axillary line and from the second to the seventh intercostal nerve on the parasternal area. While several studies have shown that intravenous anesthesia leads to a lower incidence of postoperative nausea and vomiting [11–13], it is worth noting that anesthesia with propofol and remifentanil tends to be more expensive compared to alternative methods [14, 15]. Another intriguing study by Jabs et al. [16] assesses the integration of general anesthesia with intraoperative infiltration of a standard tumescent solution (1 L Ringer’s lactate, 50 mL 1% lidocaine, and 1 mL epinephrine 1:1000) into the predetermined pocket area of each breast prior to dissection. In their retrospective analysis of breast augmentation conducted under general anesthesia, the researchers observed a decrease in postoperative pain and a notable reduction in the need for postoperative pain medication during the immediate perioperative period. However, it is important to note that the use of general anesthesia still extends the postoperative recovery time. As tumescent local anesthesia infiltration typically takes around 20 min and can be conducted outside the operating room, patients can undergo preparation in a monitoring environment while not yet in the OR. Sub-muscular implant placement required approximately 740 mL of solution per breast. Nevertheless, these quantities remain well within safety limits; indeed, the established safe doses of TLA in the adult population go up to 55 mg/kg [17, 18]. Lidocaine toxicity is closely associated with its plasma levels, which can be influenced by excessively rapid systemic uptake, impaired liver metabolism, or drug interactions. Therefore, monitoring the patient during infiltration is crucial for ensuring safety. Avoiding the use of propofol, ketamine or fentanest reduces the risk of drug-related complications such as respiratory depression, hypo- or hypertension, bradycardia and nausea or emesis. In addition to that, the recovery time after surgery is short. This compensates for the longer preparing time before surgery for performing local anesthesia and allowing it to act. It is extremely important to strictly select the candidates for this procedure, meeting the criteria for ASA status I or II [19]. We strongly advise the presence of an anesthesiologist during the operation, for continuous monitoring of oxygen saturation, and checking the patient’s respiratory and cardiocirculatory status. It is also advisable to perform these procedures where it is possible to immediately convert to general anesthesia in case of need, even if we never experienced it in our case series. For the infiltration of tumescent solution beneath the muscle, it is recommended to insert a 2-mm-diameter cannula beneath the pectoralis major muscle, directly superficial to the ribs and chest wall [20]. However, this approach warrants consideration of the potential risk of pneumothorax. In our practice, we partially expose the superficial aspect of the muscle before cannula insertion, facilitating a clear understanding of the thorax shape and exact rib position. During this surgical step, it is advisable to proceed with caution, especially as some resistance from the muscular fibers may be encountered while inserting the cannula. Another crucial aspect is initiating the injection of tumescent solution slowly, with manual assessment by placing a hand over the breast, ensuring proper flow in the correct plane beneath the muscle. The sub-muscular implant positioning involves cutting the pectoralis major muscle insertions at the ribs and sternum level dissecting the pocket with a combination of blunt dissection and cautery. Despite the absence of muscle relaxants, maintaining muscular tone has not been a significant concern during surgery [21]. This procedure induces bleeding by severing the perforating branches of the internal thoracic artery and vein [22]. Tumescent local anesthesia, utilizing epinephrine, induces vasoconstriction, thereby minimizing blood loss and bleeding throughout the surgery [23]. This facilitates clearer visibility for the surgeon and simplifies the creation of the pocket under the muscle. However, it is crucial to meticulously address hemostasis after the cessation of the epinephrine effect during surgery to prevent postoperative bleeding. Successful hemostasis eliminates the need for drains, reducing patient discomfort and averting a potential cause of implant infection. Notably, we documented only one case of hematoma (5%) and no cases of seroma formation, a complication rate comparable to the literature on sub-muscular breast augmentation [24–26], regardless of the anesthesia technique employed. Tumescent solution also aids in accurate plane identification and elevation of the pectoralis major muscle through sub-muscular hydro-dissection. Given that the injected solution volume influences breast shape modification, precise preoperative markings for implant placement are crucial. It is important to inform the patient that the breasts may appear swollen in the initial postoperative weeks. We observed only one instance of implant dislocation (5%). The comprehensive analysis of outcomes, detailing both major and minor complications, adds depth to the discussion by providing a realistic perspective on the procedure’s efficacy. Complication rates associated with breast augmentation are minimal, regardless of whether the surgery is conducted in cisgender or transfeminine patients [27]. Recently, Cuccolo et al. [28] conducted a retrospective analysis of the ACS NSQIP database from 2005 to 2017, categorizing breast augmentation patients into cisgender and transfeminine cohorts, and observed low complication rates of 1.8% and 1.6%, respectively, over the 30-day postoperative period, with no statistically significant difference between the two groups (*P *= 0.890). The enduring complications of breast implantation in transfeminine individuals mirror those in cisgender patients, encompassing issues such as hematoma, seroma, symmastia, capsular contracture, diminished breast or nipple sensation, dystrophic scar formation, and implant migration. In anecdotal instances, corrective procedures in transfeminine patients pose challenges due to the presence of already thin breast tissue and limited skin coverage. Overall, implant-based breast augmentation is deemed a secure procedure. While instances of patient regret after breast augmentation are infrequent, they do occur sporadically, even though no cases of regret occurred in our case series. Factors contributing to post-procedural regret may include unfavorable surgical outcomes, procedural complications, and a lack of social support from family and partners [29]. In our study, while a 5% major complication rate was recorded, the overall minor complication rate of 10% further supports the relatively manageable nature of the issues observed, such as implant dislocation and dystrophic scar formation. Understanding these occurrences in the context of the procedure’s overall success rate and patient satisfaction is crucial in evaluating the procedure’s overall efficacy. Our study not only contributes to the technical aspects of the surgery, but also significantly advances the discourse surrounding transgender healthcare, emphasizing the importance of tailored surgical approaches that not only deliver physical changes but also uphold the emotional and psychological well-being of the patients [30, 31]. In his editorial comment [32], Latham explains how transsexual patients desire aesthetic surgeries to ‘‘look normal’’ and ‘‘enhance beauty’’ [33] just as other patients do, only their requests are ‘‘cross-gendered. In a prospective, noncomparative, cohort study, Weigert et al [34] suggested that the gains in breast satisfaction, psychosocial well-being, and sexual well-being after male-to-female transsexual patients undergoing breast augmentation are statistically significant and clinically meaningful to the patient at 4 months after surgery and in the long term. In our experience with sub-muscular breast implant positioning using TLA, we obtained fully satisfied patients. One notable limitation of this study is the relatively small sample size of patients included in the research, which may affect the generalizability of the findings to a broader population. The study relied primarily on data collected through questionnaires and self-reported aesthetic outcomes from the participants. While these self-reported measures offer valuable insights into the subjective experiences of the patients, they also introduce the potential for response bias and may not fully capture the nuanced complexities of the aesthetic outcomes under investigation. Furthermore, the reliance on self-reported data poses a challenge in terms of objective verification, potentially introducing a degree of subjectivity into the results. As such, caution should be exercised when extrapolating the study’s conclusions beyond the specific cohort studied, and future research with larger and more diverse participant groups, as well as objective measurements statistic analysis, is warranted to enhance the robustness and applicability of the findings. By acknowledging the role of these procedures in alleviating gender dysphoria and fostering the desired physical attributes, our study underscores the broader impact on individuals’ quality of life and mental well-being. The emphasis on informed decision-making, extensive research, and consultation with experienced healthcare professionals in transgender care further elevates the discussion, highlighting the necessity for a comprehensive and supportive approach to such interventions.

## Conclusion

Overall, augmentation mammaplasty emerges as a valuable therapeutic modality for transgender patients aspiring to augment their physical femininity and alleviate gender dysphoria. In tandem with this clinical decision-making process, it is imperative for individuals to meticulously appraise the procedural intricacies, comprehensively grasp the potential hazards and advantages, and solicit counsel from proficient healthcare practitioners specialized in transgender healthcare. Tumescent Local Anesthesia stands out as a secure and efficacious method for executing breast augmentation surgery with implant placement in sub-muscular anatomical position. Patients have expressed satisfaction with the technique, with no intraoperative complications documented. Nevertheless, we still recommend the presence of a board-certified anesthesiologist for correct selection of patients and contingency planning in the event of substantive anesthesia-related complications.
